# Exploring real-world unmet clinical needs among patients on first-line treatment with HER2-negative advanced gastric, gastroesophageal junction or esophageal adenocarcinoma in Asia, Europe and the United States

**DOI:** 10.3389/fonc.2026.1863920

**Published:** 2026-10-19

**Authors:** Naureen Starling, Jaffer Ajani, Kohei Shitara, Jin Gu, Shivani K. Mhatre, Cosmina Hogea, Daniel O. Koralek, Chloe Hammond, Annabel Lambert, Florian Lordick

**Affiliations:** 1 Royal Marsden Hospital, London and Surrey, London, United Kingdom; 2 MD Anderson Center, Houston, TX, United States; 3 National Cancer Center Hospital East, Kashiwa, Chiba, Japan; 4 Gilead Sciences, Inc, Foster City, CA, United States; 5 Adelphi Real World, Bollington, United Kingdom; 6 Department of Medicine II, University of Leipzig Medical Center, Cancer Center Central Germany, Leipzig, Germany

**Keywords:** biomarkers, concordance, HER2-negative, immune checkpoint inhibitors, PD-L1, stomach cancer, treatment patterns

## Abstract

**Introduction:**

With a rapidly evolving treatment landscape for gastric cancer, gastroesophageal junction cancer, and esophageal adenocarcinoma (GC/GEJC/EAC), there is a need to understand how novel treatments are implemented and remaining unmet clinical needs in real-world clinical practice. This study sought to identify unmet clinical needs by describing biomarker testing, first-line (1L) treatment patterns, physician-reported real-world treatment response, physician-patient side-effect concordance and its association with HRQoL in HER2-negative GC/GEJC/EAC patients in real-world clinical practice.

**Methods:**

Data were drawn from the Adelphi Real World GC Disease Specific Programme™ (DSP), a cross-sectional survey of physicians and their HER2-negative GC/GEJC/EAC patients, conducted in Asia, Europe and the United States (US) between October 2022−May 2024. Physicians reported patient demographics, biomarker testing, treatment patterns, physician-reported real-world treatment response, and side-effects, while patients reported side-effects and completed the EQ-VAS. Patients were grouped by region, Programmed Death Ligand-1 (*PD-L1)* combined positive score (CPS), and 1L treatment received. Kappa analysis assessed physician-patient side-effect concordance and linear regressions assessed associations between side-effect concordance and EQ-VAS scores. Other analyses were descriptive.

**Results:**

Data were drawn for 1623 patients on 1L treatment (Asia: n=492, Europe: n=977, US: n=154) and 643 (Asia: n=226, Europe: n=377, US: n=40) on second line plus (2L+) treatment. Median (IQR) age was 67.0 years (59.0, 71.0) and 70% consulted in an academic setting. *PD-L1* testing rates were high (Asia: 81%, Europe: 86%, US:87%). Most patients with CPS score <5 received chemotherapy (CT) at 1L, while 57% with CPS ≥5 received CT+immune checkpoint inhibitors (ICI). Physicians reported that 47% of patients receiving 1L CT and 38% 1L CT+ICI experienced side-effects, and that 48% and 51% of those on 2L+ therapy achieved a complete real-world response. Side-effects were more commonly reported by patients than physicians, with poor−slight concordance reported for most side-effects. An association between physician-patient concordance on shortness of breath and EQ-VAS scores was observed.

**Conclusions:**

Biomarker testing, treatment patterns, real-world treatment response, and physician-patient concordance suggest an unmet clinical need to improve biomarker-guided treatment optimization, access to treatments, timely implementation of new evidence, and physician-patient communication regarding side-effects and HRQoL.

## 1 Introduction

Stomach and esophageal cancers, including gastric cancer, gastroesophageal junction cancer, and esophageal adenocarcinoma (GC/GEJC/EAC), accounted for 7.5% of new cancer cases and 11.4% of cancer-related deaths globally in 2022 (1). Outcomes are often poor despite treatment advances, with 5-year survival rates between 20 and 40% in most countries (2). While overexpression of human epidermal growth factor receptor-2 (HER2) has historically been associated with more aggressive tumors and poorer clinical outcomes, the advent of HER2-targeted treatments options over the past decade has significantly improved prognosis in this population (3). In contrast, treatment options for HER2-negative patients have remained limited with suboptimal outcomes (4–6).

For patients with advanced or metastatic HER2-negative GC/GEJC/EAC, chemotherapy (CT) was traditionally the first-line (1L) of systemic treatment given, most commonly doublet platinum/fluoropyrimidine-based CT (7, 8). Treatment options have expanded in the last five years with the approval of several new immunotherapies for use in HER2-negative GC/GEJC/EAC by global regulatory bodies including the United States (US) Food and Drug Administration (FDA), the European Commission, the National Medical Products Administration in China (NMPA), the Pharmaceutical and Medical Device Agency (PMDA) in Japan, and the Ministry of Food and Drug Safety (MFDS) of the Republic of Korea (9–18). Novel treatment options include the programmed cell death protein 1 (PD-1) immune checkpoint inhibitors (ICI) nivolumab, pembrolizumab, and tislelizumab, and the claudin 18.2 (*CLDN-18.2*) monoclonal antibody zolbetuximab (19–21). Of these, only nivolumab was approved by the NMPA and PMDA for 1L use in HER2-negative advanced or metastatic GC/GEJC/EAC at the time of this study. Nivolumab, pembrolizumab, and tislelizumab demonstrated clinically meaningful efficacy in clinical trials, with all three improving overall survival (OS) in combination with CT compared to CT-alone or placebo in patients with *PD-L1* expression (22, 23). ICIs are recommended for use in advanced and metastatic HER2-negative GC/GEJC/EAC in regional guidelines across Asia, Europe, and the US, though combined positive score (CPS) threshold criteria varies by region (24). Zolbetuximab, which indirectly modulates immune response through *CLDN-18.2*, has also demonstrated survival benefit in clinical trials (25). Other immunotherapies that have shown promise in clinical trials include those targeting T cell immunoglobulin and immunoreceptor tyrosine-based inhibitory motif domains (26), and *FGFR2* (27).

In an evolving treatment landscape, we need to understand how novel ICIs are prescribed by physicians at 1L and how biomarker testing is used to inform treatment decisions in real-world practice. To optimize treatment, we must also understand the side-effects of ICI+CT and those associated with the established treatment option CT-alone, how perceptions of side-effects may differ between patients and physicians, and how differing perceptions are associated with a patient’s health-related quality of life (HRQoL). Given recent approvals, real-world data on ICI use is sparse. In order to identify remaining unmet clinical needs, this study described biomarker testing, first-line treatment patterns, physician-reported real-world treatment response, treatment side-effects, physician-patient concordance in side-effect reporting, and the association between concordance and HRQoL scores in patients with HER2-negative advanced or metastatic GC/GEJC/EAC. Unmet clinical needs were interpreted across three domains: biomarker-informed treatment optimization, access to treatment and implementation of novel evidence, and physician-patient communication of side-effects and HRQoL.

## 2 Methods

Data were drawn from the Adelphi Real World GC Disease Specific Programme™ (DSP), a large independent, multinational, multifaceted data source consisting of data from cross-sectional surveys (with retrospective elements) of physicians and their patients treated for GC/GEJC/EAC. These surveys were conducted in Asia (China, Japan, and South Korea), Europe (France, Germany, Spain, and the United Kingdom [UK]), and the US between October 2022−May 2024. The DSP methodology has been previously described (28, 29), validated (30), and shown to be consistent over time (31).

Physicians were recruited via local field work agents following a short screening questionnaire. To be eligible for inclusion, physicians must have been medical oncologists, radiation oncologists, gastroenterologists, or surgeons involved in the treatment and management of at least five patients with advanced or metastatic GC/GEJC/EAC per month at the time of data collection. Physicians first completed a short attitudinal survey containing questions on their attitudes towards, perceptions of, and barriers to biomarker testing. Physicians were then asked to complete a patient record form using the patients’ medical charts for their next five consecutive consulting patients aged +18 years who had received a physician-confirmed diagnosis of and who were receiving treatment for advanced or metastatic GC/GEJC/EAC on or after the first of January 2021 (**Supplementary Figure S1**). The patient record form collected data on patient demographics, retrospective biomarker testing and drug therapy (therapy given, therapy line), retrospective physician-rated real-world treatment response, and treatment side-effects.

These same patients were then asked to complete a voluntary patient self-complete form containing questions on side-effects as well as the EQ-VAS, a validated patient-reported outcome measure (32). The EQ-VAS asked patients to rate their own HRQoL on a scale from 0 (worst possible health) to 100 (perfect health).

### 2.1 Analysis

Three distinct patient groups were included in this analysis; patients on 1L treatment at survey to assess biomarker testing, treatment patterns, and side-effects, patients on 2L+ treatment to assess physician-reported real-world treatment response to completed 1L treatment, and patients on 1L treatment at survey who had a matched patient record form and self-complete form to assess side-effect concordance and HRQoL (**Supplementary Figure S1**).

For the purposes of analysis, patients were split by region (Asia, Europe, or the US), treatment received at 1L (CT or CT+ICI), and *PD-L1* combined positive score (CPS; <5 or ≥5). To ease interpretation of the different populations being analyzed, the stratifications used for each analysis are shown in **Supplementary Table S1**. A change in treatment line was defined as the end of one treatment regimen and the start of another distinct regimen. The CT treatment group consisted of patients receiving fluorouracil + leucovorin + oxaliplatin (FOLFOX) or oxaliplatin + capecitabine (CAPOX). The CT+ICI group were defined as receiving FOLFOX or CAPOX with nivolumab or pembrolizumab (tislelizumab was not approved for 1L use at the time of this survey). Other treatment groups are defined in the legend of **Figure 1**.

**Figure 1:**
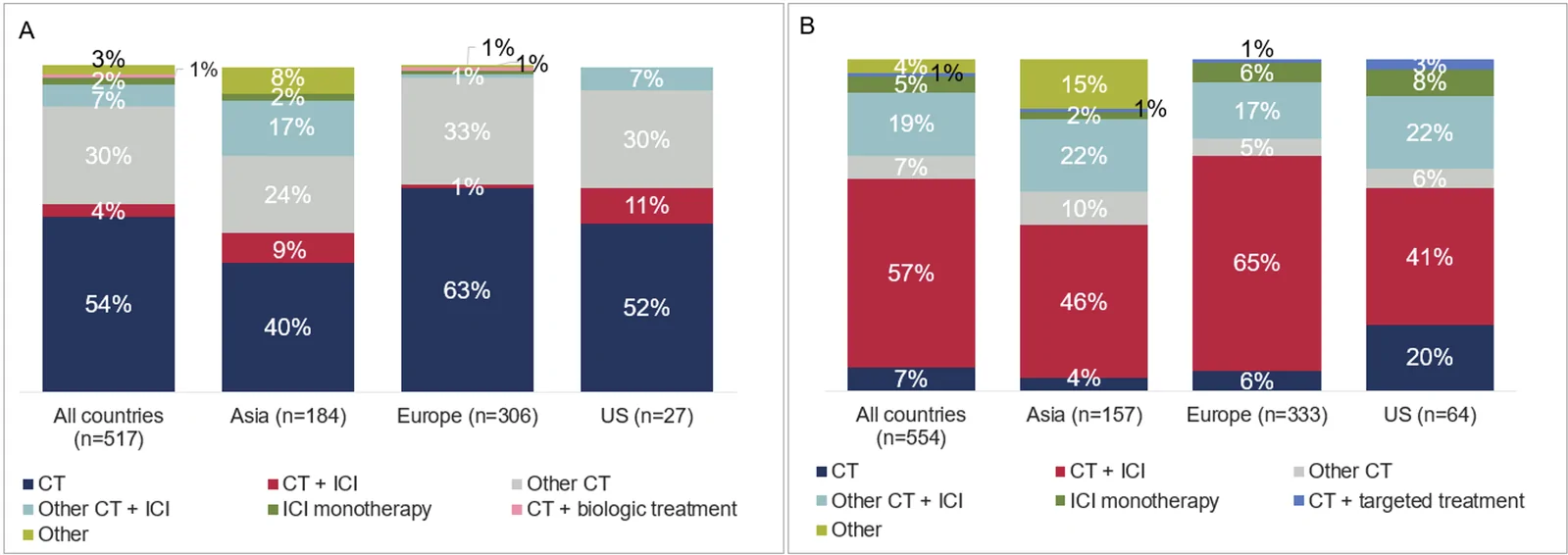
First-line treatment patterns by region among patients with a known PD-L1 combined positive score: **(A)** CPS <5, **(B)** CPS ≥5. CPS, combined positive score; US, United States; CT, chemotherapy; ICI, immune checkpoint inhibitor. Asia consisted of China, South Korea and Japan. Europe consisted of the France, Germany, Spain and the United Kingdom CT, FOLFOX or CAPOX, CT+ICI, FOLFOX or CAPOX + nivolumab or pembrolizumab, other CT, any chemotherapy combination which is not FOLFOX or CAPOX, other CT + ICI, other CT + nivolumab or pembrolizumab, ICI monotherapy = nivolumab or pembrolizumab alone, CT+ biologic, other CT + ramucirumab, CT + targeted, other CT + trastuzumab, trastuzumab-deruxtecan, entrectinib or larotrectinib, and other = any treatment not included.

*PD-L1* expression was measured using CPS score, with higher scores indicating greater expression. Patients were split by CPS <5 or ≥5 based on the recommended threshold for nivolumab prescription at 1L in advanced GC/GEJC/EAC according to contemporary guidelines at time of survey, with supplementary analysis in those with CPS >1 and >1–<5 (33). Physicians reported real-world treatment response based on information available to the treating physician and the criteria used in routine clinical practice, standardized response criteria, imaging modality, assessment timing or schedule were not provided nor were central review or independent response adjudication conducted.

No formal inferential comparisons between treatment and regional stratifications were performed as groups were purely observational and therefore differed in patient characteristics, clinical context, treatment availability, practice setting, and sample size. All outcomes are summarized and interpreted descriptively, with inferential analyses limited to the prespecified concordance analyses utilizing Cohen’s kappa statistics and linear regressions.

Concordance on side-effects was calculated as an aggregate percentage alignment for each side-effect, with each side-effect assumed to be weighted equally. Kappa values less than 0.00 were interpreted as poor agreement; 0.00–0.20 as slight agreement; 0.21–0.40 as fair agreement; 0.41–0.60 as moderate agreement; 0.61–0.80 as substantial agreement; and 0.81–1.00 as almost perfect agreement (34).

Linear regression was used to assess the association between physician-patient concordance for each side-effect and EQ-VAS scores. The linear regression model simultaneously included physician-patient concordance on the presence of nausea, diarrhea, loss of appetite, vomiting, constipation, trouble sleeping, and shortness of breath. No other covariates were included in the model. Standard errors were adjusted to account for physician clustering. All other data analysis was purely descriptive. For interpretation, unmet clinical needs were identified based on a study specific framework rather than a validated composite measure. This framework consisted of three domains: incomplete biomarker-informed treatment optimization, barriers to treatment access or implementation, and gaps in physician-patient recognition and communication of side-effects and HRQoL.

Numerical and ordinal data are presented as mean (standard deviation; SD) or median (interquartile range; IQR) as appropriate, categorical data are presented as percentage with n provided as appropriate. Missing data were not imputed, the numbers and proportion of patients with data available and unavailable for PD-L1 CPS, MSI/MMR and EQ-5D VAS are reported in **Supplementary Table S2**. All analyses were conducted in Stata (StataCorp. 2023. Stata Statistical Software: Release 18. College Station, TX: StataCorp LLC).

### 2.2 Ethics

The Adelphi Real-World GC DSP was conducted in line with European Pharmaceutical Market Research Association (EphMRA) guidelines (35)and all relevant country-specific legislation including the Health Insurance Portability and Accountability Act (HIPAA), Health Information Technology for Economic and Clinical Health Act (HiTECH), and the Declaration of Helsinki (36). The DSP protocol was submitted to the Pearl institutional review board (protocol number: AG9313) and received an ethical waiver. No personally identifiable data were collected, and data were pseudonymized by assigning a code to allow for matching between physician and patient responses. All participants provided informed consent to participate prior to data collection.

## 3 Results

### 3.1 Demographics and clinical characteristics

In total, 450 physicians (Asia: n=133, Europe: n=225, US: n=92) reported data for 1623 patients receiving 1L treatment (Asia: n=492, Europe: n=977, US: n=154) and 643 receiving 2L+ treatment (Asia: n=226, Europe: n=377, US: n=40) at time of survey. Overall, median (range) age was 67 (30–89) years and 72% of patients were male. Most patients had recurrent GC/GEJC/EAC (70%) and 83% had metastatic disease at survey. Of those with a known CPS score, 61% had CPS >1, with 48% having a CPS <5 and 52% ≥5 (**Table 1**). Half (52%) of patients in the US were treated in a community setting, 30% in Europe and 23% in Asia. In the US, 53% of patients had relapsed, 77% in Europe and 64% in Asia (**Table 2**). Data availability for PD-L1 CPS, MSI/MMR and EQ-5D VAS are summarized in **Supplementary Table S2**.

**Table 1:** Baseline demographics and clinical characteristics of patients on first-line treatment at survey, stratified by 1L treatment.

	All 1L patients	By 1L treatment		
		1L CT	1L CT+ICI	1L other
**Sample size, n**	**1623**	**551**	**389**	**683**
Median age at data collection, n (range)	67 (30-89)	67 (30-84)	65 (35-83)	67 (33-89)
<50	111 (7)	36 (7)	23 (6)	52 (8)
≥50-64	541 (33)	169 (31)	149 (38)	223 (33)
≥65	971 (60)	346 (63)	217 (56)	408 (60)
Biological sex, n (%)				
Male	1173 (72)	396 (72)	275 (71)	502 (73)
Female	450 (28)	155 (28)	114 (29)	181 (27)
Ethnicity, n (%)				
White	729 (45)	319 (58)	144 (37)	266 (39)
Non-white	92 (6)	29 (5)	27 (7)	36 (5)
Practice setting, n (%)				
Academic	1143 (70)	446 (81)	278 (71)	419 (61)
Community	480 (30)	105 (19)	111 (29)	264 (39)
Disease status at diagnosis, n (%)				
*De Novo*	474 (29)	108 (20)	68 (17)	298 (44)
Relapsed	1144 (70)	440 (80)	319 (82)	385 (56)
Stage at data collection, n (%)				
Locally advanced	277 (17)	59 (11)	25 (6)	193 (28)
Metastatic	1346 (83)	492 (89)	364 (94)	490 (72)
ECOG status at initiation of 1L treatment, n (%)				
0/1	1300 (80)	424 (77)	336 (86)	540 (79)
2/3/4	322 (20)	127 (23)	53 (14)	142 (21)
CPS score, n (%)	1071	318	336	417
<5	517 (48)	279 (88)	21 (6)	217 (52)
≥5	554 (52)	39 (12)	315 (94)	200 (48)
>1	654 (61)	92 (29)	321 (96)	241 (58)

CT, chemotherapy; CPS, combined positive score; ECOG, Eastern Cooperative Oncology Group; ICI, immune checkpoint inhibitor; 1L, first-line. Note that Ethnicity data was not collected in France or in Asia. CT = FOLFOX or CAPOX. CT+ICI = FOLFOX or CAPOX + nivolumab or pembrolizumab. Other, other treatments excluding CT or CT+ICI. The bold values refer to the sample sizes.

**Table 2:** Baseline demographics and clinical characteristics of patients on first-line treatment at survey, stratified by region.

	By region		
	Asia	Europe	US
**Sample size, n**	**492**	**977**	**154**
Median age at data collection, n (range)	65 (33-89)	67 (30-89)	66 (38-89)
<50	47 (10)	53 (5)	11 (7)
≥50-64	197 (40)	285 (29)	59 (38)
≥65	248 (50)	639 (65)	84 (55)
Biological sex, n (%)			
Male	355 (72)	702 (72)	116 (75)
Female	137 (28)	275 (28)	38 (25)
Ethnicity, n (%)			
White	–	629 (64)	100 (65)
Non-white	–	39 (4)	53 (34)
Practice setting, n (%)			
Academic	381 (77)	688 (70)	74 (48)
Community	111 (23)	289 (30)	80 (52)
Disease status at diagnosis, n (%)			
*De Novo*	174 (35)	227 (23)	73 (47)
Relapsed	315 (64)	748 (77)	81 (53)
Stage at data collection, n (%)			
Locally advanced	96 (20)	137 (14)	44 (29)
Metastatic	396 (80)	840 (86)	110 (71)
ECOG status at initiation of 1L treatment, n (%)			
0/1	411 (84)	761 (78)	128 (83)
2/3/4	81 (16)	215 (22)	26 (17)
CPS score, n (%)	341	639	91
<5	184 (54)	306 (48)	27 (30)
≥5	157 (46)	333 (52)	64 (70)
>1	195 (57)	389 (61)	70 (77)

CT, chemotherapy; CPS, combined positive score; ECOG, Eastern Cooperative Oncology Group; Asia consisted of China, South Korea and Japan. Europe consisted of France, Germany, Spain and the United Kingdom. Note that Ethnicity data was not collected in France or in Asia. The bold values refer to the sample sizes.

### 3.2 Biomarker testing prior to first-line treatment in patients on first-line treatment at survey

*PD-L1* testing rates were high at 81% in Asia (n=492), 86% in Europe (n=977) and 87% in the US (n=154), with high rates of microsatellite instability/mismatch repair immunohistochemistry (MSI/MMR IHC) testing also reported (**Figure 2A**). The most reported reasons for biomarker testing were “to inform treatment decisions”, “to assess treatment response”, or “to predict recurrence” (**Figure 2B**), while the most reported barriers to testing were “cost concerns” and “time constraints”. A quarter of physicians in the US (n=92) reported “insurance” as a barrier to biomarker testing, 4% in Europe (n=225) and 3% in Asia (n=133; **Figure 2C**).

**Figure 2:**
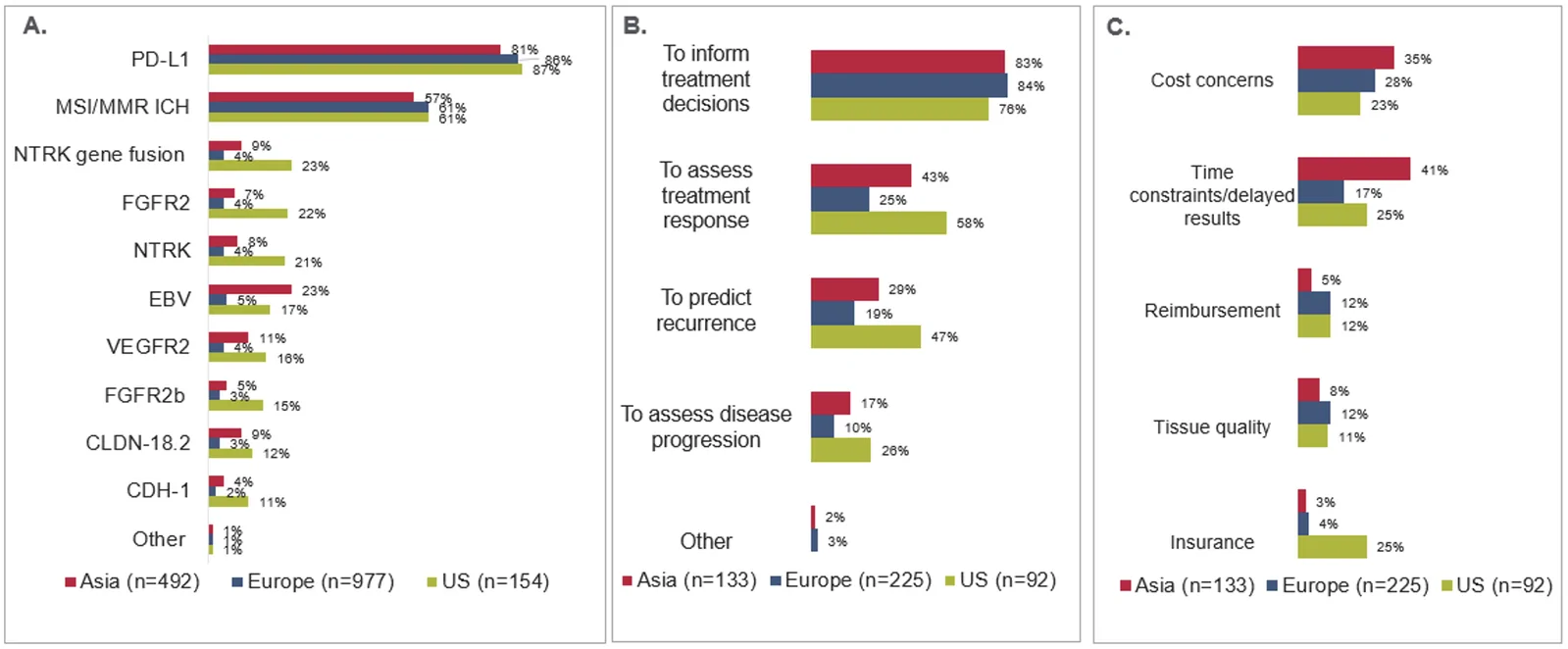
**(A)** Percentage of patients tested for biomarkers, **(B)** physician-reported reasons for biomarker testing, and **(C)** physician-reported barriers to biomarker testing. US, United States. Asia consisted of China, South Korea and Japan. Europe consisted of France, Germany, Spain, and the United Kingdom. For **(B, C)** percentages were calculated using the number of physicians who provided a response to the corresponding biomarker-testing item within each region as the denominator. Regional denominators are shown in each panel. Multiple responses were permitted; therefore,percentages may sum to more than 100%.

### 3.3 First-line treatments by *PD-L1* combined positive score and region in patients on first-line treatment at survey

Most patients (n=517) with a known CPS score of <5 were prescribed either CT (54%) or other CT-alone (30%) at 1L, while 57% of those with CPS ≥5 received CT+ICIs. A fifth (20%) of patients with CPS ≥5 in the US (n=64) received CT alone (**Figure 1**), while 29% (n=2/7) of those with CPS >1–<5 received CT+ICI. Meanwhile, 15% of patients in Asia (n=157) with CPS ≥5 received other therapies (**Figure 1**) and 10% (n=4/41) of patients with CPS >1–<5 received CT+ICI. No patients (n=0/56) in Europe with CPS >1–<5 received CT+ICIs (**Supplementary Figure S2**). Caution should be taken when interpreting CPS >1–<5 subgroup due to the low number of patients in each region, particularly in the US.

Physician-reported real-world treatment response and time to discontinuation for 1L treatment were assessed in advanced or metastatic HER2-negative patients who were on 2L+ treatment at survey (n=643; for demographics see **Supplementary Table S3**). Of these, 311 received CT at 1L and 74 CT+ICI, with 49% and 56% achieving a partial/complete real-world response at 1L, respectively. Of those with a CPS ≥5 who received CT+ICI at 1L (n=51), 57% achieved a partial/complete real-world response (**Figure 3**). Mean (SD) treatment duration for 1L treatment was 6.2 (3.2) months for CT and 7.5 (5.8) months for CT+ICI.

**Figure 3:**
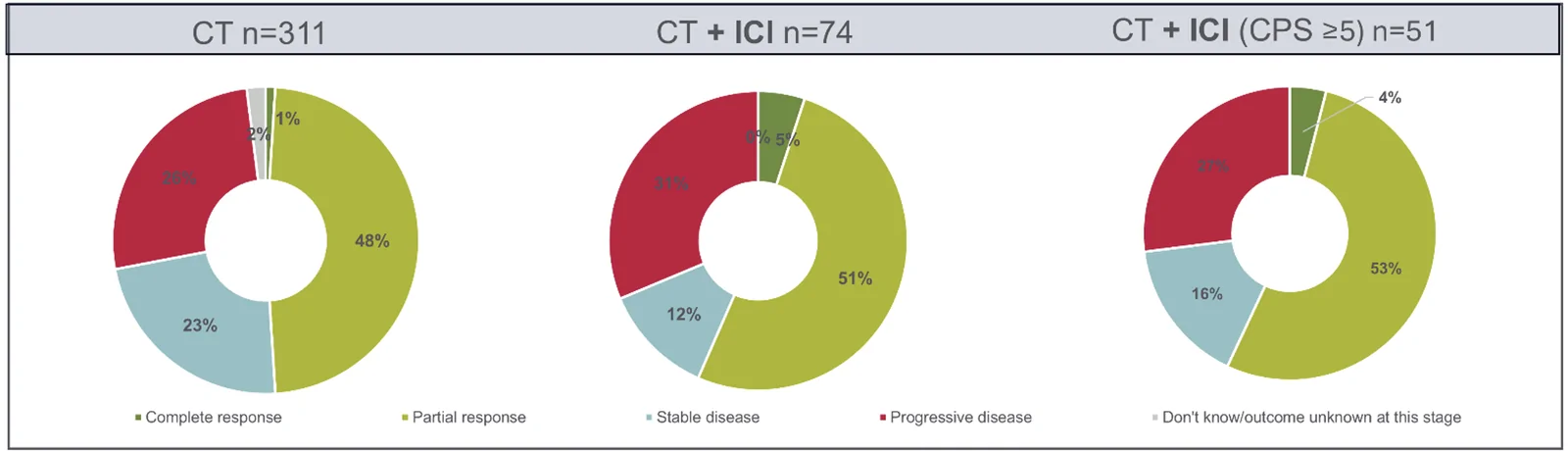
Real-world treatment response to first-line treatment regimen in patients on 2L+ treatment at survey. Data collected from second-line+ patients who have completed first-line treatment. CT. FOLFOX or CAPOX and CT+ICI , FOLFOX or CAPOX + nivolumab or pembrolizumab.

### 3.4 Side-effect concordance in patients on first-line treatment at survey

Physicians reported side-effects in 47% of patients receiving 1L CT (n=260) and 38% receiving 1L CT+ICI (n=147), with “nausea”, “peripheral neuropathy” and “fatigue” reported most frequently (**Figure 4**). Among 265 patients with matched physician and self-reported data who reported side-effects, concordance was poor to slight for most side-effects (κ: -0.012–0.177) except diarrhea (kappa: 0.357) and constipation (κ: 0.214), which showed fair agreement. Notably, 77% of patients reported trouble sleeping and 56% shortness of breath, while 6% and 3% of physicians reported these, respectively. In linear regression analyses, EQ-VAS scores were associated with the degree of physician-patient concordance on shortness of breath (R²=0.0695, p=0.004). Complete regression results are reported in **Supplementary Table S4**.

**Figure 4:**
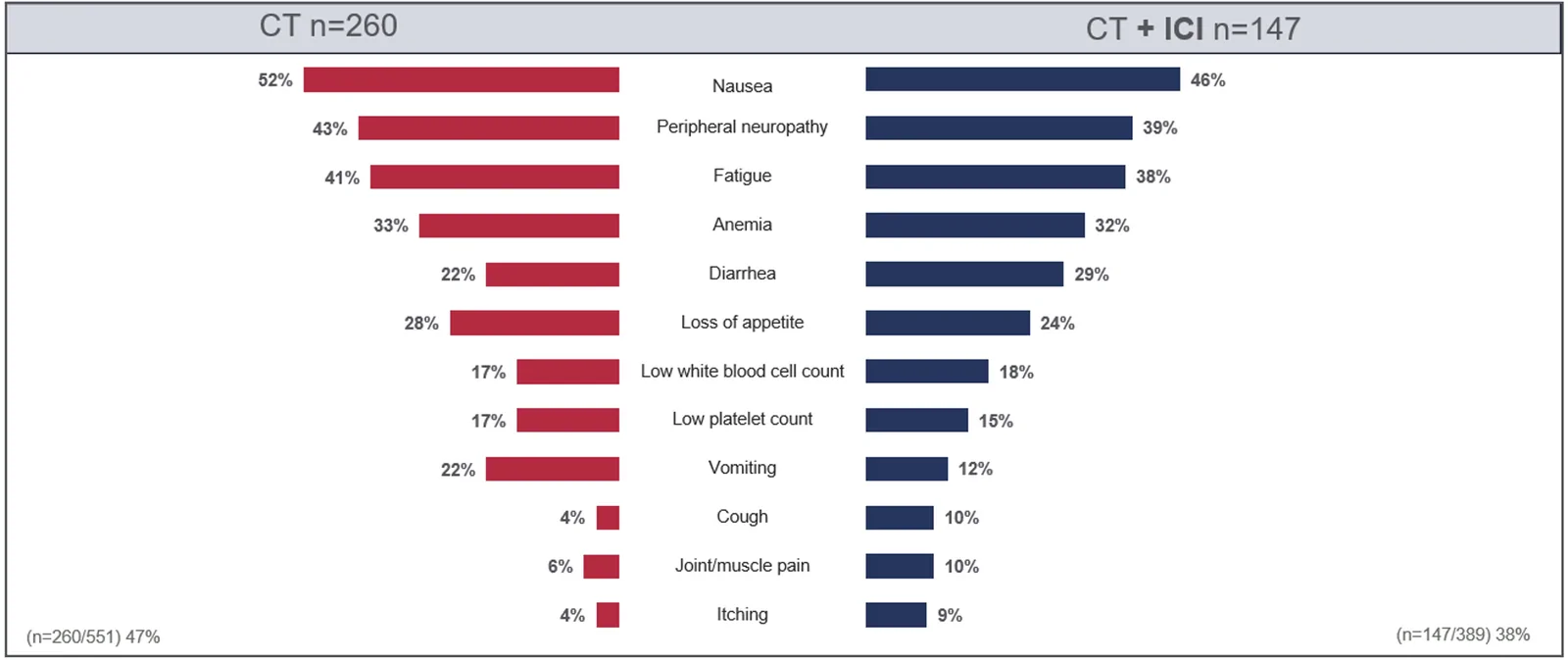
Physician-reported side-effects associated with first-line treatment by regimen. CT, Chemotherapy; ICI, Immune checkpoint inhibitor; CT, FOLFOX or CAPOX; CT+ICI, FOLFOX or CAPOX + nivolumab or pembrolizumab.

## 4 Discussion

In this large real-world study of advanced and metastatic HER2-negative GC/GEJC/EAC, we observed good uptake of *PD-L1* and MSI/MMR IHC testing, with physicians reporting using biomarker testing to inform treatment decisions. Treatment generally aligned with *PD-L1* status by region, though there were notable exceptions, particularly in the US. Physician-reported real-world treatment response and side-effect profiles were numerically similar between patients prescribed CT and CT+ICIs. Physician-patient concordance was poor-slight for most reported side-effects, with EQ-VAS scores associated with the degree of concordance on the presence of shortness of breath. Together, these findings suggest unmet clinical needs in the management of HER2-negative GC/GEJC/EAC across three domains, a need to improve sub-optimal biomarker-guided treatment optimization, overcome barriers to accessing and implementing treatment, and improve physician-patient communication regarding side-effects experienced and HRQoL.

Although PD-L1 testing rates were high, not all patients underwent testing, despite guidance in some regions recommending that all patients with advanced or metastatic GC be tested to optimize treatment (33). *PD-L1* and MSI status are known predictors of ICI response in metastatic disease, with CT being of limited benefit in patients with MSI (37–39). *PD-L1* testing is particularly key in regions with more stringent CPS thresholds associated with approvals, such as Europe, where nivolumab is approved only in those with CPS ≥5, pembrolizumab in those with CPS ≥1, and tislelizumab approved for tumor area positivity score 5% or greater (10, 12, 13). Other biomarkers were tested for less frequently, likely due to many not being actionable at time of survey. For example, the *CLDN-18.2* monoclonal antibody-based zolbetuximab was only approved in some regions towards the end of the survey period (19–21).

CPS-guided treatment prescribing may reflect differences in regulatory approvals and eligibility criteria across regions. Nivolumab and pembrolizumab were approved for use regardless of tumor PD-L1 status in the US, unlike in Europe where approval was based on CPS threshold, which could explain why CT+ICIs were broadly prescribed across CPS categories in the US (10–12, 14, 24). Despite strong uptake of CT+ICIs, almost a quarter of patients with CPS ≥5 did not receive CT+ICI. Whilst this could indicate that some patients were not receiving optimal treatment, it is likely that not all patients were eligible for ICIs, with many factors impacting eligibility such as performance status, comorbidity and age (40). However, these results suggest an unmet clinical need to improve biomarker-guided treatment optimization to ensure eligible patients with high CPS scores receive guideline-recommended therapy.

Similarly, regional treatment patterns may reflect differences in practice setting within this sample or regional access to treatments. A high proportion of patients in the US sample were seen in community practices where uptake of immunotherapy has typically been lower compared to in academic settings across a range of cancers (41–43). Furthermore, during the survey period, nivolumab was the only 1L ICI approved across all regions, while approval and availability of pembrolizumab and incorporation into local guidelines varied by country and over time (14, 24, 44). Together, these findings suggest regional barriers remain to treatment access and to the timely implementation of evolving evidence across healthcare systems and practice settings.

Though physician-reported real-world treatment response observed here for 1L CT+ICI was broadly consistent with estimates reported in selected clinical trials and real-world studies (45–47), response cannot be directly compared as criteria for real-world response, imaging requirements, and assessment timings were not standardized, nor was response assessment centrally reviewed or aligned with Response Evaluation Criteria in Solid Tumors (RECIST). Though the proportion of patients experiencing side-effects was numerically similar between CT+ICI and CT-alone subgroups, this should be interpreted cautiously, as side-effects and adverse events are often underrecognized and underreported in routine clinical practice (48, 49). Side-effect profiles may also reflect increasing clinician experience and improved management of immune-related side-effects over time (50, 51). Nevertheless, immune-related adverse events remain an important consideration with ICI treatment (52–55), highlighting the need for therapies that improve outcomes while minimizing treatment burden.

Finally, our findings suggest there is a need for improved communication between physicians and patients about the side-effects of treatment experienced. Side-effects were more frequently reported by patients, with concordance regarding shortness of breath associated with EQ-VAS scores. Maintaining HRQoL is one of the key aims of treatment reported by cancer patients, assigned equal value to length of life (56). To minimize the most burdensome side-effects, provide appropriate support to manage disease and treatment burden and improve HRQoL, physicians must first recognize side-effects and the burden they represent. Side-effects not only impact the patients physical and functional wellbeing, but their mental wellbeing too, suggesting that the support needs of patients may go beyond simply treating side-effects (57). Implementation of standardized patient-reported outcome measures in clinical practice could help to improve communication, having previously been shown to improved symptom control, HRQoL, and reduce the need for hospitalization/unplanned healthcare episodes in real-world clinical practice (58).

## 5 Limitations

Though the DSP collected a robust multinational sample of HER2-negative patients with GC/GEJC/EAC, there are some standard limitations associated with the DSP methodology that should be considered when interpreting results. As this was a pragmatic sample, when splitting the data some sample sizes were low (such as when splitting by CPS score in the US) precluding statistical analysis, meaning any differences are purely descriptive and may not be significant.

Though physicians were asked to provide data for consecutive eligible patients to avoid discretional selection bias, physician participation was influenced by willingness to engage. Minimal patient selection criteria were implemented, however eligible patients must have been alive, actively consulting, and receiving treatment at time of survey, with only those that progressed to 2L+ included in completed-1L physician-reported real-world response analysis. These selection criteria may have affected estimates of treatment response, treatment duration, and side-effect burden, though the size and direction of this bias cannot be determined from these data.

Physicians were asked to report real-world treatment response based on their own judgment and the criteria they would use in clinical practice, no standardized response criteria, prespecified imaging modalities, uniform assessment timing, or central review were employed. Real-world response may therefore reflect a combination of radiologic assessment and clinical judgment, which may introduce assessment variability, limiting comparison between physicians, regions, and published RECIST-based clinical-trial results. A limited number of patients were recruited from each physician to limit the impact of any one physicians judgment on the sample and to enrich for commonalities in practices. Finally, questions asking about side-effects were worded slightly differently for patients and physicians to aid patient understanding, so assumptions had to be made when comparing; for example, when comparing “feelings of worry” (patient-reported) with “anxiety” (physician-reported).

## 6 Conclusions

Using a multinational real-world dataset, this study explored three domains of unmet clinical need in HER2-negative GC/GEJC/EAC: biomarker-informed treatment optimization, treatment access and implementation, and physician-patient communication regarding symptom burden and HRQoL. PD-L1 and MSI/MMR IHC testing was frequently reported, although treatment patterns varied across CPS categories and regions, highlighting the challenges of implementing biomarker-guided care in routine practice. The descriptive response and side-effect findings reinforce the need for therapies that improve outcomes while minimizing treatment burden. Finally, physician-patient discordance in side-effect reporting suggests an opportunity to improve side-effect recognition and communication through standardized patient-reported outcome measures. Future work is needed to develop new treatments and identify novel actionable biomarkers beyond *PD-L1* to improve treatment response rates and patient outcomes in advanced and metastatic HER2-negative GC/GEJC/EAC patients. Furthermore, research is needed to develop new, more effective methods of collecting side-effect and impact data from patients to provide adequate support and improve patient HRQoL.

## Data Availability

The data analyzed in this study is subject to the following licenses/restrictions: The Adelphi Real World Gastric Cancer Disease Specific Programme (DSP) is an independent survey conducted by Adelphi Real World. The DSP methodology and data set is a wholly owned product of Adelphi Real World that is subscribed to by multiple pharmaceutical companies, of which Gilead is one. This publication was supported by Gilead Sciences, Inc. Requests to access these datasets should be directed to https://adelphirealworld.com/.
