# Supplementary material for: Exploring real-world unmet clinical needs among patients on first-line treatment with HER2-negative advanced gastric, gastroesophageal junction or esophageal adenocarcinoma in Asia, Europe and the United States
Source: Front Oncol. 2026 Oct 19;16:1863920. doi: 10.3389/fonc.2026.1863920 (PMC13724567; doi:10.3389/fonc.2026.1863920)
Supplement: Supplementary file 1 [file SupplementaryFile1.docx]

# 13 Supplementary Figures

**Supplementary Figure S1:** Sample attrition

**^Abbreviations:^** ^GC - Gastric cancer; GEJ - gastroesophageal junction cancer; EAC - esophageal adenocarcinoma; US - United States; 1L - first-line; tx - treatment; 2L -second-line^

**Supplementary Table S1**: Stratifications used in each analysis

| **Stratifications** | **Biomarker testing** | **1L treatment patterns** | **Real-world treatment response** | **Side-effects experienced** | **Side-effect concordance** | **Impact of concordance on HRQoL** |
| --- | --- | --- | --- | --- | --- | --- |
| **Patient population** | Patients on 1L treatment only | | Patients on 2L+ | Patients on 1L treatment only | Patients on 1L treatment with matched physician and patient data | |
| **Country** | Overall, Asia, Europe, and US | | Overall only | | | |
| **Treatment** | Overall only | CT only, CT + ICI, other CT, other CT + ICI, ICI monotherapy, CT + biologic, CT + targeted, and other | Overall only | | Overall | |
| **CPS score** | Overall only | <5, ≥5, >1, and >1−<5 | Overall and ≥5 (CT+ICI only) | Overall only | | |

**^Abbreviations:^** ^1L - first-line; HRQoL - Health-related Quality of Life; 2L+ - second-line plus treatment; US - United States; CT - chemotherapy; ICI - immune checkpoint inhibitor; CPS - combined positive score. Asia consisted of China, South Korea and Japan. Europe consisted of France, Germany, Spain and the United Kingdom. CT = FOLFOX or CAPOX. CT+ICI = FOLFOX or CAPOX + nivolumab or pembrolizumab^

**Supplementary Table S2:** Proportions of unknown or missing data for first-line patients

| **Outcome** | **All regions** |
| --- | --- |
| **Sample size, n** | **1623** |
| **PD-L1, n (%)** | |
| Not tested | 253 (16) |
| Test performed but CPS score unknown | 299 (22) |
| **MSI/MMR, n (%)** | |
| Not tested | 649 (40) |
| Test performed but result unknown | 20 (2) |
| **Patient-reported sample size, n** | **265** |
| **EQ-VAS score, n (%)** | |
| Missing data/unknown result | 2 (1) |

**^Abbreviations:^** ^PD-L1 – programmed death-ligand 1; CPS – combined positive score; MSI/ MMR - Microsatellite Instability/ Mismatch Repair^

**Supplementary Figure S2**: First-line treatments by region for patients with PD-L1 score for patients with **A) A)** CPS >1, and **B)** CPS >1−<5

**^Abbreviations:^** ^CPS - combined positive score,^ ^US - United States; CT – chemotherapy; ICI - immune checkpoint inhibitor. Asia consisted of China, South Korea and Japan. Europe consisted of the France, Germany, Spain and the United Kingdom. CT = FOLFOX or CAPOX, CT+ICI = FOLFOX or CAPOX + nivolumab or pembrolizumab, other CT = any chemotherapy combination which is not FOLFOX or CAPOX, other CT + ICI = other CT + nivolumab or pembrolizumab, ICI monotherapy = nivolumab or pembrolizumab alone, CT+biologic = other CT + ramucirumab, CT+targeted = other CT + trastuzumab, trastuzumab-deruxtecan, entrectinib or larotrectinib, and other = any treatment not included above^

**Supplementary Table S3:** Baseline demographics and clinical characteristics of patients on second-line treatment at survey who had received prior first-line treatment

|  | **All regions** | **By 1L treatment** | | | **By region** | | |
| --- | --- | --- | --- | --- | --- | --- | --- |
|  |  | **1L CT** | **1L CT+ICI** | **1L Other** | **Asia** | **Europe** | **US** |
| **Sample size, n** | **643** | **311** | **74** | **258** | **226** | **377** | **40** |
| **Median age at data collection (range), n (%)** | 68  (35-86) | 69  (38-85) | 66  (35-81) | 67  (37-86) | 66  (35-86) | 69  (38-86) | 63.5  (45-79) |
| <50 | 49 (8) | 16 (5) | 9 (12) | 24 (9) | 30 (13) | 17 (5) | 2 (5) |
| ≥50–64 | 184 (29) | 77 (25) | 27 (36) | 80 (31) | 70 (31) | 95 (25) | 19 (48) |
| ≥65 | 410 (64) | 218 (70) | 38 (51) | 154 (60) | 126 (56) | 265 (70) | 19 (48) |
| **Biological sex, n (%)** | | | | | | | |
| Male | 417 (65) | 202 (65) | 56 (76) | 159 (62) | 143 (63) | 247 (66) | 27 (68) |
| Female | 226 (35) | 109 (35) | 18 (24) | 99 (38) | 83 (37) | 130 (34) | 13 (32) |
| **Ethnicity, n (%)** | | | | | | | |
| White | 268 (42) | 155 (50) | 20 (27) | 93 (36) | - | 241 (64) | 27 (68) |
| Non-white | 30 (5) | 16 (5) | 6 (8) | 8 (3) | - | 18 (5) | 12 (30) |
| **Practice setting, n (%)** | | | | | | | |
| Academic | 466 (72) | 244 (78) | 58 (78) | 164 (64) | 185 (82) | 264 (70) | 17 (42) |
| Community | 177 (28) | 67 (22) | 16 (22) | 94 (36) | 41 (18) | 113 (30) | 23 (58) |
| **Disease status at diagnosis, n (%)** | | | | | | | |
| De Novo | 228 (35) | 77 (25) | 11 (15) | 140 (54) | 94 (42) | 111 (29) | 23 (58) |
| Relapsed | 415 (65) | 234 (75) | 63 (85) | 118 (46) | 132 (58) | 266 (71) | 17 (43) |
| **Stage at data collection, n (%)** | | | | | | | |
| Locally advanced | 85 (13) | 15 (5) | 1 (1) | 69 (27) | 34 (15) | 40 (11) | 11 (28) |
| Metastatic | 558 (87) | 296 (95) | 73 (99) | 189 (73) | 192 (85) | 337 (89) | 29 (73) |
| **ECOG status at data collection, n (%)** | | | | | | | |
| 0/1 | 394 (61) | 148 (65) | 226 (60) | 20 (50) | 190 (61) | 58 (78) | 146 (57) |
| 2/3/4 | 249 (39) | 78 (35) | 151 (40) | 20 (50) | 121 (39) | 16 (22) | 112 (43) |
| **CPS score, n (%)** | **378** | **165** | **64** | **149** | **169** | **189** | **20** |
| <5 | 226 (60) | 133 (81) | 11 (17) | 82 (55) | 96 (57) | 125 (66) | 5 (25) |
| ≥5 | 152 (40) | 32 (19) | 53 (83) | 67 (45) | 73 (43) | 64 (34) | 15 (75) |
| >1 | 218 (58) | 68 (41) | 56 (88) | 94 (63) | 94 (56) | 106 (56) | 18 (90) |

**^Abbreviations:^** ^CT - chemotherapy; CPS - combined Positive Score; ECOG - Eastern Cooperative Oncology Group; ICI - immune checkpoint inhibitor; IQR – interquartile range, 2L+ - second line and beyond. Asia consists of China, South Korea and Japan. Europe consisted of France, Germany, Spain, and the United Kingdom. Note that Ethnicity data was not collected in France or in Asia. CT = FOLFOX or CAPOX and CT+ICI = FOLFOX or CAPOX + nivolumab or pembrolizumab, and other =all other treatments excluding CT or CT+ICI.^

**Supplementary Table S4:** Regression analysis showing how physician-patient concordance on key side-effects impacts patient-reported outcomes across all regions

| **Quality of life measurements (outcome variables)** | **All patient side-effects** | | | |
| --- | --- | --- | --- | --- |
|  | **Coefficient with EQ- VAS score** | **Patient-reported** | **Physician-reported** | **Kappa score** |
| **Nausea** | -2.186 | 79% | 49% | 0.1228 |
| **Diarrhea** | 1.045 | 47% | 31% | 0.3569 |
| **Loss of appetite** | 1.369 | 86% | 28% | -0.0123 |
| **Vomiting** | -2.082 | 53% | 22% | 0.1769 |
| **Constipation** | 1.075 | 37% | 8% | 0.2139 |
| **Trouble sleeping** | 3.718 | 77% | 6% | 0.0257 |
| **Shortness of breath** | 6.031* | 56% | 3% | 0.0473 |

**^Abbreviations^**^: Significant associations are highlighted by *.^
